# Preoperative Multiparametric Magnetic Resonance Imaging Predicts Biochemical Recurrence in Prostate Cancer after Radical Prostatectomy

**DOI:** 10.1371/journal.pone.0157313

**Published:** 2016-06-23

**Authors:** Richard Ho, Mohummad M. Siddiqui, Arvin K. George, Thomas Frye, Amichai Kilchevsky, Michele Fascelli, Nabeel A. Shakir, Raju Chelluri, Steven F. Abboud, Annerleim Walton-Diaz, Sandeep Sankineni, Maria J. Merino, Baris Turkbey, Peter L. Choyke, Bradford J. Wood, Peter A. Pinto

**Affiliations:** 1 Urologic Oncology Branch, National Cancer Institute, National Institutes of Health, Bethesda, Maryland, United States of America; 2 Molecular Imaging Program, National Cancer Institute, National Institutes of Health, Bethesda, Maryland, United States of America; 3 Laboratory of Pathology, National Cancer Institute, National Institutes of Health, Bethesda, Maryland, United States of America; 4 Center for Interventional Oncology, National Cancer Institute, National Institutes of Health, Bethesda, Maryland, United States of America; 5 Department of Surgery, Division of Urology, University of Maryland, Baltimore, Maryland, United States of America; National Health Research Institutes, TAIWAN

## Abstract

**Objectives:**

To evaluate the utility of preoperative multiparametric magnetic resonance imaging (MP-MRI) in predicting biochemical recurrence (BCR) following radical prostatectomy (RP).

**Materials/Methods:**

From March 2007 to January 2015, 421 consecutive patients with prostate cancer (PCa) underwent preoperative MP-MRI and RP. BCR-free survival rates were estimated using the Kaplan-Meier method. Cox proportional hazards models were used to identify clinical and imaging variables predictive of BCR. Logistic regression was performed to generate a nomogram to predict three-year BCR probability.

**Results:**

Of the total cohort, 370 patients met inclusion criteria with 39 (10.5%) patients experiencing BCR. On multivariate analysis, preoperative prostate-specific antigen (PSA) (p = 0.01), biopsy Gleason score (p = 0.0008), MP-MRI suspicion score (p = 0.03), and extracapsular extension on MP-MRI (p = 0.03) were significantly associated with time to BCR. A nomogram integrating these factors to predict BCR at three years after RP demonstrated a c-index of 0.84, outperforming the predictive value of Gleason score and PSA alone (c-index 0.74, p = 0.02).

**Conclusion:**

The addition of MP-MRI to standard clinical factors significantly improves prediction of BCR in a post-prostatectomy PCa cohort. This could serve as a valuable tool to support clinical decision-making in patients with moderate and high-risk cancers.

## Introduction

Prostate cancer (PCa) is the leading noncutaneous cancer in men in the United States, responsible for nearly 30,000 deaths and 230,000 new cases per year [1]. Radical prostatectomy (RP) is an established treatment for localized disease [2]. However, rates of biochemical recurrence (BCR) after RP are reportedly as high as 27% with two-thirds of BCR occurring within two years after RP [3,4]. Furthermore, BCR has been associated with progression to distant metastases and cancer-specific mortality [5]. Several studies have attempted to identify predictors of BCR after RP but with limited accuracy [6]. Thus, a more reliable method to predict BCR would be clinically useful in treatment decision-making.

Tools such as the Kattan nomogram and Han tables have enabled clinicians to use clinical parameters such as pretreatment prostate-specific antigen (PSA), clinical stage, and biopsy Gleason score to predict the probability of BCR [7,8]. Attempts to increase the accuracy of these models with additional clinical parameters have produced limited added benefit [9]. Magnetic resonance imaging (MRI) has been recognized as an excellent modality to stage and localize PCa due to its exceptional soft-tissue contrast and high spatial resolution [10]. Several studies have explored the utility of prostate MRI in predicting BCR but with varied results [11,12].

Advances in multiparametric MRI (MP-MRI), consisting of T2-weighted (T2W), diffusion-weighted (DW), and dynamic contrast enhanced (DCE) imaging have improved detection and localization of clinically significant PCa [13,14]. Although MP-MRI has been extensively studied for its diagnostic capabilities, its significance in predicting postoperative outcomes is less well understood [15,16]. A predictive model for the likelihood of recurrence after treatment, combining standard clinical factors with imaging results, could be a valuable tool for patients and clinicians. Therefore, we evaluated the performance of preoperative MP-MRI characteristics in predicting BCR following RP.

## Materials and Methods

### Patient selection, assessment, treatment, and follow-up

**Fig 1:**
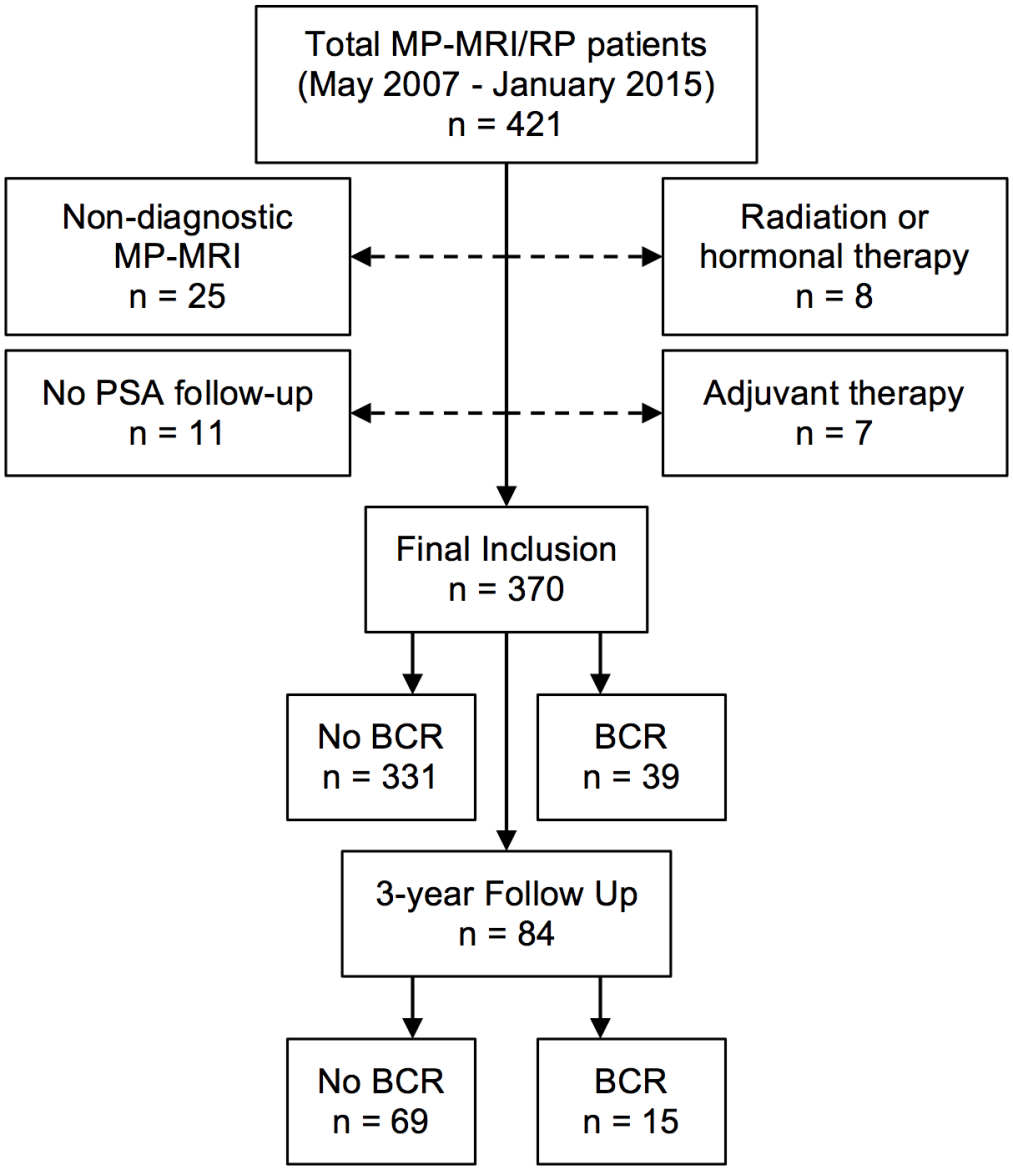
Flowchart of patient selection. MP-MRI = multiparametric magnetic resonance imaging; PSA = prostate-specific antigen; RP = radical prostatectomy.

Patients were enrolled under an institutional review board (IRB) approved (ClinicalTrials.gov: NCT00102544), prospective trial with all data collection and follow-up performed in accordance with the United States Health Insurance Portability and Accountability Act. Patients provided written informed consent with approval of the consent procedure by the IRB. From May 2007 to January 2015, 421 consecutive patients underwent MP-MRI followed by robotic-assisted RP at a single institution (Fig 1). Patients were excluded if they had no recorded PSA values postoperatively, had non-diagnostic MRI (e.g. hemorrhage, hip prosthesis, movement artifacts), received prior hormone or radiation therapy, or had adjuvant treatments before documented BCR.

All patients underwent total serum PSA screening, digital rectal exam (staging per American Joint Committee on Cancer, 7^th^ Edition), standard 12-core systematic transrectal ultrasound (TRUS) guided biopsy, as well as MP-MRI of the prostate. A subset of patients also underwent a targeted MRI/TRUS fusion guided biopsy. MP-MRI data including lesion number, total prostate volume, MP-MRI suspicion score, and MRI-based suspicion for extracapsular extension (mECE) and seminal vesicle invasion (mSVI) together with biopsy Gleason score (highest from either standard or targeted MRI/TRUS fusion guided biopsy) were obtained.

All RP procedures were performed by a single urologist (PAP) and all pathology was reviewed by a single genitourinary pathologist (MJM) with pathologic grade, stage, margin, and lymph node status noted. Follow-up protocol involved monitoring serum PSA levels at one, three, and six months after RP, with annual PSA levels subsequently. BCR was defined following the guidelines of the American Urological Association Localized Prostate Cancer Update Panel report [17] as a serum PSA ≥ 0.2 ng/ml with a confirmatory value of ≥ 0.2 ng/ml, a single PSA ≥ 0.4 ng/ml, or by receipt of salvage therapy specifically due to an increasing postoperative PSA.

### Imaging protocol

Diagnostic MP-MRI was performed on a 3.0 Tesla MRI scanner (Achieva, Philips Healthcare, Best, Netherlands) with a 16-channel cardiac surface coil (SENSE, Philips Healthcare, Cleveland OH) positioned over the pelvis and an endorectal coil (BPX-30, Medrad, Pittsburgh, PA) as previously described [13]. The MRI protocol included T2W imaging, DW imaging with apparent diffusion coefficient mapping (ADC), and axial three-dimensional fast field echo DCE MRI sequences. These images underwent blinded centralized radiological evaluation by two radiologists (BT, PLC) with 8 and 16 years of prostate MRI experience, respectively. PCa suspicion scores (low, moderate, or high) were assigned to each lesion using previously described criteria, which have been associated with both the occurrence of PCa and tumor grade [18, 19]. The now standardized PI-RADS (Prostate Imaging Reporting and Data System) criteria were not routinely used during the time frame of this study [20]. However, a low, moderate, and high MP-MRI suspicion score is analogous to 1–2, 3–4, and 5 score, respectively using the PI-RADS system [21].

### Outcome measures and statistical analysis

Statistical analysis was performed using JMP^®^ 11.0 (SAS Institute Inc., Cary, NC) with a threshold for significance of p <0.05. Cox proportional hazards regression models were constructed for all clinical and imaging variables. Variables meeting a threshold of p <0.15 were included in the multivariate analysis. BCR-free survival (BCRFS) was estimated using the Kaplan-Meier method. Patients without recurrence were censored by their last follow-up time with survival curves compared using the log-rank test. Collinearity was assessed in the multivariate analysis by examining the effects of adding a combined term for potentially collinear covariates.

Utilizing factors found to be associated with BCR on multivariate regression, a nomogram was generated using the R statistical software package (http://www.r-project.org). A subset of 84 men from the total cohort who had complete three years of follow-up was examined to generate the nomogram with three-year BCR chosen as the outcome of interest. Stepwise analysis of the impact of each individual predictor on the c-index of the nomogram was examined and PSA was excluded as it had minimal improvement on the overall predictive ability of the nomogram. A calibration curve to assess the performance of the nomogram was generated using bootstrap analysis with n = 40 to assess predicted against actual probability of BCR from the nomogram.

## Results

Of the 421 patients who underwent RP, 370 patients met the inclusion criteria for analysis (Fig 1). In this population, 39 patients (10.5%) experienced BCR with a median time to recurrence of 14.0 months (IQR 3.7–27.5). The median follow-up time for patients with no biochemical recurrence was 22.3 months (IQR 11.9–33.9). Within the 370 patients, 206 (55.7%) underwent fusion biopsy, and 84 had complete 3-year follow up with 15 (17.9%) experiencing BCR. Preoperative patient characteristics for both cohorts are displayed in Table 1. Median patient age and preoperative PSA for the total cohort were 60 years (IQR 55–65) and 5.8 ng/ml (IQR 4.1–9.6), respectively. Post-prostatectomy variables for both cohorts including pathologic Gleason score, pathologic stage, lymph node invasion, and positive surgical margins are displayed in Table 2.

**Table 1:** Preoperative clinical and multiparametric magnetic resonance imaging characteristics of patients undergoing radical prostatectomy.

**Clinical Characteristics**		
**Median (IQR)**	**Total Cohort**	**Nomogram Cohort**
Age, yr	60 (55–65)	58 (55–63)
BMI	28 (26–31)	28 (25–31)
Preoperative PSA, ng/ml	5.5 (4.0–8.7)	5.2 (3.7–9.9)
**No (%)**		
Race		
White	240 (72.5)	54 (64.3)
Black	66 (19.9)	19 (22.6)
Other	25 (7.6)	11 (13.1)
Clinical stage		
T1c	301 (90.9)	71 (84.5)
≥ T2a	30 (9.1)	13 (15.5)
Biopsy Gleason score		
6	90 (27.2)	29 (34.5)
7	184 (55.6)	46 (54.8)
≥ 8	57 (17.2)	9 (10.7)
**MP-MRI Characteristics**		
**Median (IQR)**	**Total Cohort**	**Nomogram Cohort**
MRI prostate volume, cc	37 (30–47)	39 (28–49)
MRI lesions, no	2 (1–3)	2 (1–3)
**No (%)**		
MP—MRI suspicion score		
Low	45 (13.6)	17 (20.2)
Moderate	208 (62.8)	37 (44.1)
High	78 (23.6)	30 (35.7)
mECE		
Absent	232 (70.0)	56 (66.7)
Present	99 (30.0)	28 (33.3)
mSVI		
Absent	322 (97.3)	83 (98.8)
Present	9 (2.7)	1 (1.2)

BMI = body mass index, IQR = interquartile range, MRI = magnetic resonance imaging, MP-MRI = multiparametric magnetic resonance imaging, mECE = extracapsular extension on magnetic resonance imaging, mSVI = seminal vesicle invasion on magnetic resonance imaging, PSA = prostate-specific antigen.

**Table 2:** Postoperative pathologic patient characteristics of patients undergoing radical prostatectomy.

Postoperative Characteristics		
No. (%)	Total Cohort	Nomogram Cohort
Pathologic Gleason score		
6	38 (10.3)	11 (13.1)
7	235 (63.5)	48 (57.1)
≥ 8	97 (26.2)	25 (29.8)
Pathologic stage		
pT2a—pT2b	52 (14.0)	10 (11.9)
pT2c	243 (65.7)	53 (63.1)
pT3a	58 (15.7)	16 (19.0)
pT3b—pT4	17 (4.6)	5 (6.0)
Lymph node invasion		
Absent	352 (95.1)	77 (91.7)
Present	18 (4.9)	7 (8.3)
Margins (all)		
Not involved	322 (87.0)	70 (83.3)
Involved	48 (13.0)	14 (16.7)
Margins (≤ pT2c)		
Not Involved	269 (91.2)	55 (87.3)
Involved	26 (8.8)	8 (12.7)

Cox proportional hazards regression analyses for BCR after RP are shown in Table 3. On univariate analysis, preoperative PSA, clinical stage, biopsy Gleason score, MP-MRI suspicion score, and mECE demonstrated a significant association with time to BCR (all p <0.01). Upon multivariate analysis, preoperative PSA, biopsy Gleason score, MP-MRI suspicion score, and mECE remained significantly associated with BCR (all p <0.05). Presence of mECE (HR = 2.10, p = 0.04) and higher suspicion lesions on MP-MRI (HR = 1.97, p = 0.02) preoperatively were both associated with an increased risk of BCR after RP.

**Table 3:** Univariate and multivariate Cox proportional hazards regression model of preoperative clinical and imaging variables predicting biochemical recurrence.

Covariate	Univariate Analysis		Multivariate Analysis	
	HR (95% CI)	P Value	HR (95% CI)	P Value
Age, years	0.98 (0.94–1.02)	0.3	-	-
BMI	0.99 (0.92–1.07)	0.8	-	-
Preoperative PSA, ng/ml	1.05 (1.02–1.07)	**<0.0001**	1.04 (1.01–1.07)	**0.01**
Race				
White	1	-	-	-
Black	0.89 (0.35–1.92)	0.8	-	-
Other	1.35 (0.46–3.23)	0.6	-	-
Clinical stage	2.87 (1.37–5.61)	**0.007**	1.38 (0.63–2.90)	0.4
Biopsy Gleason score	3.76 (2.27–6.40)	**<0.0001**	2.35 (1.37–4.14)	**0.002**
MRI prostate volume, cc	0.98 (0.96–1.00)	0.06	0.98 (0.96–1.00)	0.08
MRI lesions, no	0.80 (0.59–1.07)	0.1	0.93 (0.68–1.26)	0.7
MP—MRI suspicion score	3.49 (2.02–6.39)	**<0.0001**	1.97 (1.09–3.73)	**0.02**
mECE	4.34 (2.27–8.72)	**<0.0001**	2.10 (1.05–4.40)	**0.04**
mSVI	1.85 (0.30–6.05)	0.4	-	-

BMI = body mass index; CI = confidence interval; HR = hazard ratio; PSA = prostate-specific antigen; MP-MRI = multiparametric magnetic resonance imaging; MRI = magnetic resonance imaging. Statistical significance denoted by bold face.

In order to address the influence of margin status on preoperative predictors of BCR, a subgroup Cox regression analysis was performed for patients with negative surgical margins. Of the 370 patients, 322 (87.0%) had negative surgical margins and 22 (6.8%) men in this cohort experienced BCR. On multivariate analysis, preoperative PSA, biopsy Gleason score, and mECE remained predictive of BCR (all p <0.05, Table 4), similar to as noted in the total cohort.

**Table 4:** Univariate and multivariate Cox proportional hazards regression model of clinical and imaging variables predicting biochemical recurrence: patients with negative surgical margins.

Covariate	Univariate Analysis		Multivariate Analysis	
	HR (95% CI)	P Value	HR (95% CI)	P Value
Age, years	0.99 (0.94–1.05)	0.7	-	-
BMI	1.03 (0.93–1.12)	0.5	-	-
Preoperative PSA, ng/ml	1.07 (1.04–1.09)	**<0.0001**	1.05 (1.02–1.08)	**0.003**
Race				
White	1	-	-	-
Black	0.62 (0.14–1.83)	0.4	-	-
Other	0.98 (0.16–3.43)	1	-	-
Clinical stage	3.54 (1.35–8.42)	**0.01**	1.89 (0.69–4.75)	0.2
Biopsy Gleason score	6.71 (3.26–15.28)	**<0.0001**	4.89 (2.24–11.61)	**<0.0001**
MRI prostate volume, cc	0.99 (0.96–1.01)	0.3	-	-
MRI lesions, no	0.79 (0.52–1.17)	0.3	-	-
MP—MRI suspicion score	3.81 (1.83–8.70)	**0.0002**	1.84 (0.84–4.41)	0.1
mECE	5.45 (2.30–14.29)	**0.0001**	3.37 (1.35–9.32)	**0.009**
mSVI	1.60 (0.09–7.65)	0.7	-	-

BMI = body mass index; CI = confidence interval; HR = hazard ratio; PSA = prostate-specific antigen; MP-MRI = multiparametric magnetic resonance imaging; MRI = magnetic resonance imaging. Statistical significance denoted by bold face.

**Fig 2:**
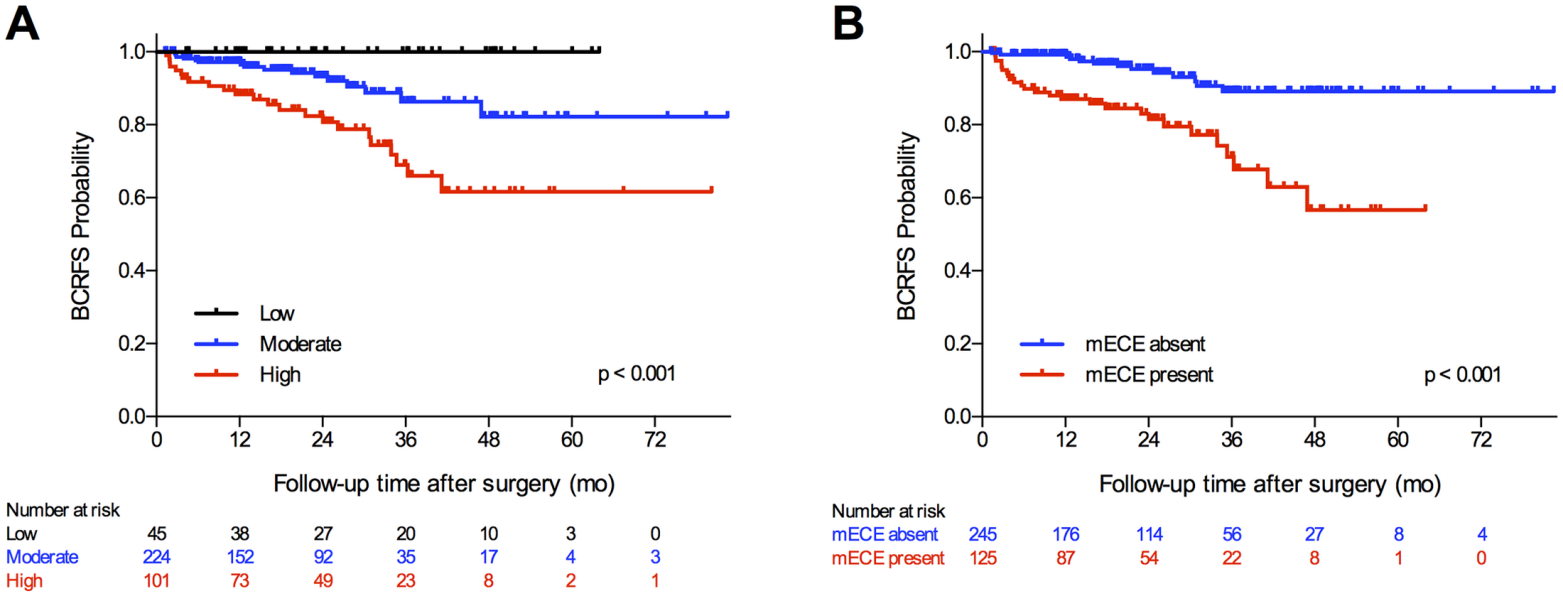
Kaplan-Meier analysis of biochemical recurrence-free survival for patients after radical prostatectomy. (A) By multiparametric magnetic resonance imaging suspicion score. (B) By extracapsular extension on magnetic resonance imaging. BCRFS = biochemical recurrence-free survival; mECE = extracapsular extension on magnetic resonance imaging; SVI = seminal vesicle invasion on magnetic resonance imaging.

To further assess the impact of imaging on prediction of BCR, Kaplan-Meier analyses were performed examining BCRFS stratified by MP-MRI suspicion score and mECE (Fig 2). BCRFS rates differed between suspicion levels and between presence of mECE (all p <0.001). The BCRFS rate for those with low MP-MRI suspicion scores remained at 100%. However, patients with high MP-MRI suspicion scores had significantly lower BCRFS than those with moderate MP-MRI suspicion scores 3 years after RP (66% vs. 86%, respectively) and 5 years after RP (62% vs. 82%, respectively). The BCRFS rate at 3 years after RP for patients with mECE was 68% compared to 89% for patients without mECE. This BCRFS difference amplified at 5 years after RP as BCRFS decreased to 57% for patients with mECE while remaining the same for patients without mECE (89%).

**Fig 3:**
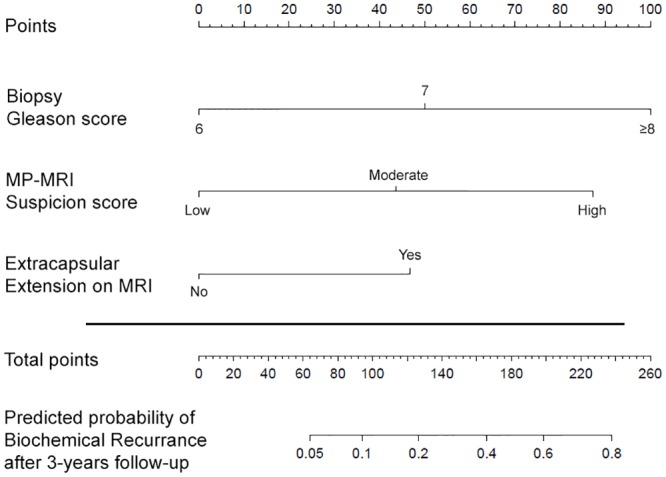
Nomogram to predict biochemical recurrence at 36 months after radical prostatectomy incorporating both clinical and multiparametric magnetic resonance imaging parameters. MP-MRI = multiparametric magnetic resonance imaging; MRI = magnetic resonance imaging.

**Fig 4:**
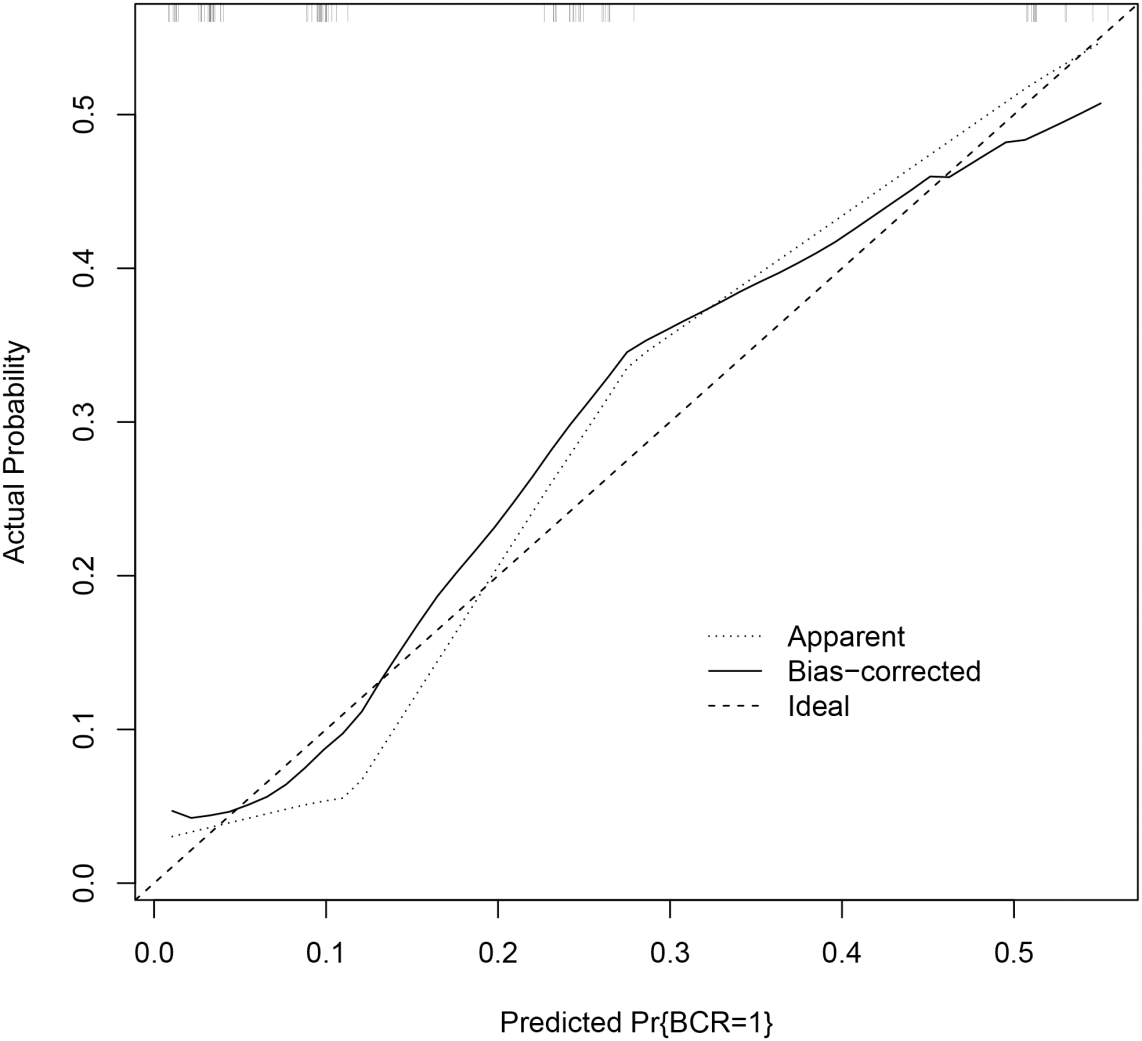
Calibration curve demonstrating performance of predicted probability of 3-year biochemical recurrence versus actual probability noted within the study. BCR = biochemical recurrence.

These findings provided sufficient evidence to support the generation of a nomogram with incorporation of pre-operative MP-MRI features for the prediction of BCR (Fig 3). The specific outcome of three-year BCR was chosen as the outcome of interest for this nomogram resulting in 84 patients being included in the analysis (Fig 1). Although PSA demonstrated a statistically significant association with BCR, the amplitude of this association was low and PSA did not exert a clinically significant impact on the prediction of BCR. Consequently, PSA was removed from this final model. The c-index of the final nomogram was 0.84. A predictive model created not including the imaging-derived features of mECE and MP-MRI suspicion score yielded a c-index of 0.74 (p = 0.02). A calibration plot demonstrated a mean absolute error of 2.9% for prediction of BCR (Fig 4). The nomogram did not consistently over-predict or under-predict throughout the entire probability range. However, it tended to under-predict in intermediate probabilities while over predicting the rate of three-year BCR at higher and lower extremes.

## Discussion

Biochemical recurrence of prostate cancer after radical prostatectomy can be a harbinger of more advanced disease and has been associated with increased rates of metastasis and prostate cancer specific mortality [22]. While RP is curative in most patients, rates of BCR following RP have been reported as high as 27% in cohorts with a median follow up of ten years [3, 23]. Thus, a preoperative predictive model for BCR could serve as an invaluable clinical tool for risk stratification and patient counseling.

MP-MRI allows for imaging-based identification and characterization of PCa, improving diagnostic accuracy [13, 14]. Yet, few studies have determined the value of MP-MRI as a non-invasive means for the prediction of BCR. Our results show that MP-MRI suspicion score and mECE, together with previously validated clinical parameters such as preoperative PSA and biopsy Gleason score, have significant added benefit in prediction of BCR after RP [7, 8].

MP-MRI suspicion score is a clinically useful method to detect and characterize PCa, especially in patients with Gleason ≥ seven, which comprised 90% of this cohort [24]. Patients who harbor highly suspicious lesions as well as those with mECE are nearly twice as likely to experience BCR, as compared to patients with only moderately suspicious lesions or those without mECE. No patients in this study with a low MP-MRI suspicion score experienced BCR, suggesting that low MP-MRI suspicion in the setting of RP corresponds to long-term BCRFS. In addition, mECE remains a significant predictor of BCR even when accounting for surgical margins, which was a confounder in previous studies [25].

Preoperative predictive models for prostate cancer recurrence have been a previously explored area of study, producing widely recognized tools such as the Kattan nomogram and the Han tables [7,8]. Imaging factors such as lesion suspicion or mECE have been associated with BCR [26–28]. However, few studies have explored the incorporation of preoperative MP-MRI variables into predictive models. Poulakis et al. showed that an artificial neural model combining 1 T pelvic-coil MRI findings with preoperative clinical variables was superior to both the Kattan nomogram and Han tables in predicting recurrence [11]. A study by Nishida et al. showed that inclusion of MRI staging significantly increased the predictive value for BCR (AUC = 0.79) in comparison to the Han tables alone (AUC = 0.67, p = 0.047) [15]. The MP-MRI nomogram we have developed is unique in its ability to incorporate clinical parameters with both MP-MRI as a biomarker (MRI suspicion score) and anatomic staging (mECE) to reliably predict BCRFS after RP in the preoperative setting.

Predictive models for BCR can serve as a crucial tool for managing patient expectations on the possibility of receiving adjuvant therapy, especially when RP is viewed as a curative measure in most patients. Also, many secondary treatments can have negative implications for quality of life [29,30]. Of the 39 patients with BCR in this cohort, a majority of these patients (85%) went on to receive salvage therapy, including external beam radiation therapy as well as androgen deprivation therapy. A nomogram developed from our predictive model was reliable (c-index = 0.84) in predicting these cases of BCR. With further validation, this MP-MRI based nomogram may be useful in risk stratification of patients before RP as well as managing patient expectations on the possibility of adjuvant interventions.

Many of our patients (55.7%) went on to receive a targeted MRI-TRUS fusion biopsy, which could be a potential confounder in this study. In order to control for this, additional analysis was performed incorporating a covariate in the multivariate analysis of fusion biopsy. This variable was not associated with BCR on univariate or multivariate analysis. Furthermore, addition of this covariate did not change the outcome of the multivariate analysis. Since MRI-TRUS fusion guided biopsies typically detect more clinically significant disease and less clinically insignificant disease, the biopsy results could also assist in the accuracy of the nomogram [31].

A limitation of this study is that it is a single institution retrospective study. Also, our MP-MRI images were performed on a single MRI unit, mostly by a single MR technologist, and read by genitourinary radiologists experienced with prostate imaging. This would all tend to improve the quality of the MRI and hence its value. The sensitivity and specificity of mECE for pathologic ECE was 58.5% and 73.3% respectively. Although significant associations were demonstrated with MRI findings consistent with ECE and biochemical recurrence, the imaging modality continues to have much room for improvement in the actual prediction of pathologic ECE. Our median follow-up time of 22.3 months is relatively short, though prediction of early BCR is clinically meaningful as nearly two-thirds of recurrences occur within two years of RP [4]. Lastly, our nomogram was generated from 84 patients with 15 (17.9%) experiencing BCR, which may limit the power and robustness of this clinical tool. As we continue with our work, we hope to increase the sample size and thus applicability of the MP-MRI based nomogram.

Additional future work will concentrate on validating the nomogram in a multicenter or prospective cohort, which could support its future clinical use. Furthermore, while the NIH suspicion score system has been validated, the incorporation of the PI-RADS system may enhance the utility of this study and is expected to perform in a similar manner.

In conclusion, the addition of MP-MRI parameters to standard clinical factors better predicts BCR in a post-prostatectomy PCa cohort. Presence of mECE and higher suspicion lesions on preoperative MP-MRI were associated with nearly twice the risk of BCR after RP. When validated, the MP-MRI based nomogram could serve as a helpful tool to support clinical decision-making.
